# Risk Factors for Mortality in Neonatal Gastric Perforation: A Retrospective Cohort Study

**DOI:** 10.3389/fped.2021.652139

**Published:** 2021-05-13

**Authors:** Yao Huang, Qi Lu, Nan Peng, Li Wang, Yan Song, Qin Zhong, Peng Yuan

**Affiliations:** ^1^Department of Neonatology, Children's Hospital of Chongqing Medical University, National Clinical Research Center for Child Health and Disorders, Ministry of Education Key Laboratory of Child Development and Disorders, Chongqing Key Laboratory of Pediatrics, Chongqing, China; ^2^Department of Neonatal Surgery, Children's Hospital of Chongqing Medical University, Chongqing, China

**Keywords:** gastric perforation, mortality, cohort study, thrombocytopenia, hyperlactatemia, shock, neonate

## Abstract

**Background:** Neonatal gastric perforation is a rare but life-threatening issue. The aim of this study was to describe the clinical characteristics and prognosis of patients with neonatal gastric perforation and identify predictive factors for poor prognosis.

**Methods:** This was a retrospective cohort study of patients with neonatal gastric perforation treated in a tertiary pediatric public hospital between April 2009 and October 2020. The enrolled patients were divided into survival and non-survival groups. Demographic information, clinical characteristics, laboratory and imaging features, and outcomes were collected from the electronic medical record. Univariate and multivariate logistic regression analyses were performed to obtain the independent factors associated with death risk. Additionally, we separated this population into two groups (pre-term and term groups) and explored the mortality predictors of these two groups, respectively.

**Results:** A total of 101 patients with neonatal gastric perforation were included in this study. The overall survival rate was 70.3%. Seventy-one (70.3%) were pre-term neonates, and sixty-two (61.4%) were low-birth-weight neonates. The median age of onset was 3 days (range: 1–11 days). Abdominal distension [98 (97.0%) patients] was the most common symptom, followed by lethargy [78 (77.2%) patients], shortness of breath [60 (59.4%) patients] and vomiting [34 (33.7%) patients]. Three independent mortality risk factors were identified: shock (OR, 3.749; 95% CI, 1.247–11.269; *p* = 0.019), serum lactic acid > 2.5 mmol/L (5.346; 1.727–16.547; *p* = 0.004) and platelet count <150 × 10^9^/L (3.510; 1.115–11.053; *p* = 0.032). There was a borderline significant association between sclerema neonatorum and total mortality (4.827; 0.889–26.220; *p* = 0.068). In pre-term infants, serum lactic acid > 2.5 mmol/L and platelet count <150 × 10^9^/L remained independent risk factors for death. In term infants, the incidence of shock, coagulopathy, pH < 7.3, serum lactic acid > 2.5 mmol/L, and hyponatremia were statistically different between non-survival and survival groups.

**Conclusion:** Shock, hyperlactatemia, and thrombocytopenia are independently associated with an increased risk of death in patients with neonatal gastric perforation. Identification of modifiable risk factors during the critical periods of life will contribute to the development of effective prevention and intervention strategies of neonatal gastric perforation.

## Introduction

Neonatal gastric perforation (NGP) is a rare but fatal disease (1–7). In 1825, Siebold reported the first case of neonatal gastric perforation (8). Duran et al. reported the mortality rate of NGP was as high as 60% (9). Despite its low incidence, NGP has dire consequences for children's development. Even when infants survive the disease, different sequelae can potentially threaten their health, including developmental delay, iron-deficiency anemia, and steatorrhea (fatty stools) (10). Most of the discussions about this disease focused on the etiology to prevent NGP and improve the prognosis. The etiology of NGP is still controversial, but severe infections, low birth weight (LBW), hypoxia, especially pre-maturity are considered as contributing factors (4, 11–14).

In recent years, some studies have focused on searching for independent factors which impact on prognosis for patients with NGP. Up to now, some studies have found that clinical characteristics and laboratory parameters may have significance in predicting the clinical deterioration of NGP. Pre-mature birth, low birth weight, gender, hyponatremia (serum sodium < 130 mEq/L), leukopenia, and metabolic acidosis (pH < 7.3) have been suggested to be unfavorable prognostic factors (15–17). However, the relative importance of these possible factors is still unclear and controversial. The evidence base was somewhat limited by the small sample size. Additionally, few studies have separately explored prognostic factors for NGP in pre-term infants and full-term infants. Therefore, to ensure the appropriate treatments are performed, it is necessary to further identify clinical features and prognostic factors of NGP.

In this retrospective study, we reviewed all patients with NGP who underwent surgery at a pediatric tertiary hospital during an 11-year period. We try to describe the overall clinical findings of NGP and investigate the possible prognostic factors with outcomes.

## Materials and Methods

### Study Design and Participants

This retrospective single-center cohort study was conducted in the Neonatal Intensive Care Unit (NICU) and Department of Pediatric Surgery at Children's Hospital of Chongqing Medical University. This is a 1,500-bed tertiary teaching hospital in Chongqing, China, ranked among the top three domestic children's hospitals (rank list: http://top100.imicams.ac.cn/home). Neonates who were surgically confirmed to have gastric perforations between April 2009 and October 2020 were included in the current study. All patients received standard of care therapy. This study received approval from the ethics committee of Children's Hospital of Chongqing Medical University. The data are anonymous, and the requirement for informed consent was therefore waived.

In the present study, we enrolled pre-term and full-term infants with NGP. Patients were divided into the survival and non-survival groups depending on vital status at hospital discharge.

### Inclusion and Exclusion Criteria

We tried to explore the prognostic factors of gastric perforation that occurred in the neonatal period, so we only included patients with onset in the neonatal period. Surgery is the most accurate way to know the perforation sites, the number of perforations, and the amount of ascites, so we only included patients who had undergone a surgical procedure. Patients were first screened for eligibility based on the following criteria: (1) Age at onset ≤ 30 d; (2) Gastric perforation was found during the operation.

Exclusion criteria: (1) The treatment before entering our hospital is unknown; (2) Neonates with major congenital structural or chromosomal anomalies; (3) Neonates without complete records of gestational age, birth weight, or blood routine.

### Data Collection

Clinical and demographic information was obtained from the medical data platform of Children's Hospital of Chongqing Medical University by trained staff using standardized data collection and quality control procedures, which produced reliable data for analysis. For each patient, the study data were recorded in an electronic case-report form. We retrospectively collected baseline clinical and biological characteristics including sex, gestational age, birth weight, mode of delivery, Apgar scores, history of perinatal asphyxia, age of onset, main symptoms and signs, pre-operative laboratory findings and imaging features, surgical intervention time (time from onset to any surgical intervention), sites of perforation, amount of ascites, number of perforations, surgical procedures, and complications. All patients were followed up until death or hospital discharge.

Pre-operative laboratory findings included leukocyte count, hemoglobin, platelet count, C-reactive protein, procalcitonin, pH, serum lactic acid, and sodium concentration.

### Definitions

Fever was defined as an axillary temperature of at least 37.3°C. In this study, sepsis and shock were defined according to Pediatric Sepsis Consensus (PSC) criteria (18). Procalcitonin (PCT) requires age adjustment, according to the cut-off point with time as previous study (19). We use the age-specific cut-off values to define high PCT level. Coagulopathy was defined as platelet count <100,000/mm^3^ or INR >1.5 or activated prothrombin time >60 s (20). Thrombocytopenia was defined as a platelet count <150 × 10^9^/L (20). Hyponatremia was defined as a serum sodium concentration (Na^+^) <130 mEq/L (20).

### Statistical Analysis

Descriptive statistics included frequency analysis (percentages) for categorical variables and means and standard deviations (SD) or medians and interquartile ranges (IQRs) for continuous variables. Comparisons were determined by Student's *t*-test or Mann-Whitney *U*-test for continuous variables as appropriate and by the use of the χ^2^-test or Fisher exact-test for categorical variables. Univariate and multivariate logistic regression were performed to explore the association of potential predictors and unfavorable outcomes. Baseline variables that were considered clinically relevant or showed a univariate relationship with outcome were entered into multivariate logistic regression models. Missing data that exceeded 10% for any variable were not considered in the multivariable logistic analysis. As such, perforation sites and PCT were excluded from the final analysis because the missing data exceeded 10%. We classified the patients into two groups (pre-term infants and term infants) based on their gestational weeks in additional analyses. Variables for inclusion were carefully chosen, given the number of events available, to ensure parsimony of the final models. The statistical significance level was set at 0.05 (two-tailed). Statistical analysis was carried out with SPSS 25.0 for Windows software (SPSS Inc., Chicago, IL, USA).

## Results

### Clinical Data

This report included 101 cases of surgically diagnosed neonatal gastric perforation between April 2009 and October 2020. In this cohort, the overall mortality rate was 29.7%. The majority of these patients were male. Seventy-one (70.3%) were pre-term neonates, and sixty-two (61.4%) were low-birth-weight neonates. The median age of onset was 3 days (range: 1–11 days). The most frequent presentations at admission were abdominal distension, followed by lethargy, shortness of breath, and cyanosis among the total population (Table 1). Except for feeding intolerance, fever, and abdominal erythema that were more frequently present in the non-survival group than the survival group, other kinds of symptoms were similar in the two groups.

**Table 1:** Demography and clinical presentation of patients.

	**Total**	**Non-survivors**	**Survivors**	***p*-value**
	**(*n* = 101)**	**(*n* = 30)**	**(*n* = 71)**	
Male gender, *n* (%)	73 (72.3)	25 (83.3)	48 (67.6)	0.107
GA (wk)^a^	35.23 (2.87)	35.20 (2.797)	35.24 (2.920)	0.785
Pre-term birth, *n* (%)	71 (70.3)	21 (70.0)	50 (70.4)	0.966
BW (g)^a^	2,323 (631)	2,316 (718)	2,324 (601)	0.228
LBW, *n* (%)	62 (61.4)	20/30 (66.7)	42 (59.2)	0.479
Cesarean section, *n* (%)	77 (76.2)	20 (66.7)	57 (80.3)	0.142
1-min Apgar^b^	9 (8–10)	9 (7.25–10)	9 (8–10)	0.722
5-min Apgar^b^	10 (9–10)	10 (9–10)	10 (9–10)	0.926
Primegravidity, *n* (%)	32 (32.0)	12 (40.0)	20 (28.6)	0.262
Primipara, *n* (%)	43 (43.0)	17 (56.7)	26 (37.1)	0.071
Feeding before onset, *n* (%)	73 (72.3)	20 (67.7)	53 (74.6)	0.561
History of perinatal asphyxia, *n* (%)	41 (40.6)	13 (43.3)	28 (39)	0.716
Age of onset (d)^b^	3 (2–4)	3.5 (1.75–5)	3 (2–4)	0.160
**Symptom**, ***n*** **(%)**				
Abdominal distension	98 (97.0)	29 (96.7)	69 (97.2)	0.889
Lethargy	78 (77.2)	24 (80.0)	54 (76.1)	0.666
Shortness of breath	60 (59.4)	16 (53.3)	44 (62.0)	0.172
Cyanosis	52 (51.5)	14 (46.7)	38 (53.5)	0.529
Vomiting	34 (33.7)	14 (46.7)	20 (28.2)	0.072
Feeding intolerance	23 (22.8)	11 (36.7)	12 (16.9)	0.007
Fever	11 (10.9)	7 (23.3)	4 (5.6)	0.024
Bloody stool	6 (5.9)	3 (10.0)	3 (4.2)	0.508
**Signs**, ***n*** **(%)**				
Absent bowel sounds	77 (76.2)	23 (76.7)	54 (76.1)	0.947
Prominent abdominal veins	43 (42.6)	14 (46.7)	29 (40.8)	0.589
Abdominal erythema	24 (23.8)	12 (40.0)	12 (16.9)	0.013

*IQR, interquartile range; GA, Gestational age; LBW, Low Birth weight*.

a *Mean and standard deviation*.

b *Median and interquartile range*.

### Laboratory Findings and Imaging Features

The laboratory findings and imaging features are shown in Table 2. Metabolic acidosis and elevated serum lactic acid was statistically associated with mortality of NGP. Compared to the patients in the survival group, those in the non-survival group underwent more frequent and more severe electrolyte disturbance, as sodium concentration was significantly lower in the patients of a dismal prognosis.

**Table 2:** Laboratory findings and imaging features of patients.

	**Total**	**Non-survivors**	**Survivors**	***p*-value**
	**(*n* = 101)**	**(*n* = 30)**	**(*n* = 71)**	
**Laboratory findings**				
WBC count, 10^9^cells/L^a^	7.40 (3.53)	4.44 (10.73)	9.17 (7.76)	0.017
<5 or >20, *n*(%)	41 (40.6)	17 (56.7)	24 (33.8)	0.033
Hb, g/L^a^	159.35 (30.30)	162.87 (34.91)	157.85 (28.27)	0.974
PLT count, 10^9^cells/L^b^	197 (141–269)	155.5 (109–197)	230 (155–281)	0.001
Thrombocytopenia	31 (30.7)	14 (46.7)	17 (23.9)	0.024
CRP>8 mg/L, *n* (%)	50/100 (50.0)	16/30 (53.3)	34/70 (48.6)	0.663
Higher PCT level, *n*(%)	54/80 (67.5)	18/25 (72.0)	36/55 (65.5)	0.562
pH^a^	7.26 (0.210)	7.13 (0.255)	7.30 (0.210)	<0.0001
pH <7.3, *n* (%)	55/95 (58)	23/29 (80)	32/66 (48)	0.005
Lac, mmol/L^b^	2.1 (1.3–3.9)	4.3 (2.4–7.45)	1.6 (1.2–2.7)	<0.0001
Lac > 2.5 mmol/L, *n* (%)	42/95 (44.2)	22/29 (75.9)	20/66 (30.3)	<0.0001
SC, mEq/L^a^	135.629 (4.950)	133.816 (5.781)	136.470 (4.304)	0.013
Hyponatremia, *n* (%)	15/100 (15.0)	9/30 (30.0)	6/70 (8.6)	0.015
**Imaging features**				
Pneumoperitoneum, *n* (%)	97 (96.0)	30 (100.0)	67 (94.4)	0.574

*WBC, White blood cell; Hb, Hemoglobin; PLT, Platelet; CRP, C-reactive protein; PCT, procalcitonin; Lac, lactic acid; SC, Sodium concentration*.

a *Mean and standard deviation*.

b *Median and interquartile range*.

### Surgical Findings and Operative Procedures

The most common perforation site was at the curvature major in the cases with accurate recording of perforation sites, followed by the curvature minor and the anterior wall of the stomach. However, no statistically significant difference was found between the two groups (Table 3).

**Table 3:** Surgical findings and operative procedures.

	**Total**	**Non-survivors**	**Survivors**	***P-*value**
	**(*n* = 101)**	**(*n* = 30)**	**(*n* = 71)**	
Surgical intervention time, h				0.782
≤ 24 h, *n* (%)	68 (67.3)	19 (63.3)	47 (66.2)	
>24 h, *n* (%)	33 (32.7)	11 (36.7)	24 (33.8)	
Perforation site, *n* (%)				0.964
Curvatura major	28 (27.7)	8 (26.7)	20 (28.2)	
Curvatura minor	13 (12.9)	3 (10.0)	10 (14.1)	
Anterior wall	14 (13.9)	5 (16.7)	9 (12.7)	
Posterior wall	3 (3.0)	1 (3.3)	2 (2.8)	
Unspecified	43 (42.6)	13 (43.3)	30 (42.3)	
Amount of ascites (ml)^a^	80 (50–150)	150 (65–200)	100 (50–150)	0.003
<100, *n* (%)	41 (40.6)	8 (26.7)	33 (46.5)	0.064
≥100, *n* (%)	60 (59.4)	22 (73.3)	38 (53.5)	
Multiple perforations, *n* (%)	18 (17.9)	8 (26.7)	10 (14.1)	0.131
Surgical procedures, *n* (%)				0.119
Gastrorrhaphy+gastrostomy	85 (84.2)	25 (83.3)	60 (84.5)	
Gastrorrhaphy+drainage	14 (13.9)	3 (10.0)	11 (15.5)	
Drainage	2 (2.0)	2 (6.7)	0 (0)	

a *Median and interquartile range*.

The incidences of multiple perforations and the amount of ascites in the non-survival group were higher than those in the survival group, but there was no statistical difference.

### Complications and Causes of Perforation

Pneumonia was the most frequently observed complication, followed by coagulopathy, sepsis, and respiratory failure. Shock, sepsis, and sclerema neonatorum were more common in the non-survival group (all *p* < 0.05) (Table 4).

**Table 4:** Complications and causes of perforation.

	**Total**	**Non-survivors**	**Survivors**	***p*-value**
	**(*n* = 101)**	**(*n* = 30)**	**(*n* = 71)**	
**Complications**, ***n*** **(%)**				
Sepsis	59 (58.4)	23 (76.7)	36 (50.7)	0.016
Pneumonia	87 (86.1)	24 (80.0)	63 (88.7)	0.398
Respiratory failure	39 (38.6)	11 (36.7)	28 (39.4)	0.794
Shock	38 (37.6)	20 (66.7)	18 (25.4)	<0.001
Coagulopathy	64 (63.4)	23 (76.7)	41 (57.7)	0.071
NEC	27 (26.7)	11 (36.7)	16 (22.5)	0.143
SN	11 (10.9)	8 (26.7)	3 (4.2)	0.003
**Cause of the perforation**, ***n*** **(%)**				
Only gastric wall muscular defects	21 (20.0)	5 (16.7)	16 (22.5)	0.507
Other causes	80 (79.2)	25 (83.3)	55 (77.5)	

*NEC, necrotizing enterocolitis; SN, sclerema neonatorum*.

### Predictors of Mortality

In univariable analysis, the odds of poor prognosis were higher in patients with sepsis, shock, sclerema neonatorum, leukocyte count <5 × 10^9^/L or >20 × 10^9^/L, thrombocytopenia, pH < 7.3, serum lactic acid > 2.5 mmol/L, and hyponatremia (Table 5).

**Table 5:** Risk factors associated with mortality.

	**Univariable OR**	***p*-value**	**Multivariable OR**	***p-*value**
	**(95% CI)**		**(95% CI)**	
Primipara	2.323 (0.953–5.662)	0.064		
Sepsis	3.194 (1.151–8.866)	0.026		
Shock	5.476 (2.131–14.07)	<0.0001	3.749 (1.247–11.269)	0.019
SN	8.000 (1.924–32.959)	0.004	4.827 (0.987–26.220)	0.068
WBC count <5 or >20 × 10^9^/L	2.833 (1.153–6.961)	0.021		
Thrombocytopenia	2.917 (1.162–7.323)	0.026	3.510 (1.115–11.053)	0.032
pH <7.3	4.073 (1.469–11.294)	0.007		
Lac > 2.5 mmol/L	7.229 (2.661–19.639)	<0.0001	5.346 (1.727–16.547)	0.004
Hyponatremia	5.490 (1.647–18.305)	0.006		
Amount of ascites ≥100 ml	2.187 (0.848–5.642)	0.105		

*CI, confidence interval; OR, odd ratio; SN, sclerema neonatorum; WBC, white blood cell; Lac, lactic acid*.

We included 95 patients with complete data for all variables (29 patients survived and 66 dead) in the multivariable logistic regression model. We found that shock, sclerema neonatorum, thrombocytopenia were associated with increased odds of death (Table 5). Sclerema neonatorum had only marginal statistical significance.

### Subgroup Analysis for Pre-Term and Full-Term Infants

We classified the patients with complete data into two groups (pre-term infants and term infants) based on their gestational weeks and explored the mortality predictors of these two groups, respectively.

Patient demographic characteristics and presentation are summarized in Table 6. Whether in the pre-term or the term group, the most common presentations at admission were abdominal distension, lethargy, shortness of breath, and cyanosis (Table 6).

**Table 6:** Demography and clinical presentation of pre-term and full-term infants.

	**Pre-term group**			**Term group**		
	**Non-survivors**	**Survivors**	***p-*value**	**Non-survivors**	**Survivors**	***p-*value**
	**(*n* = 20)**	**(*n* = 48)**		**(*n* = 9)**	**(*n* = 18)**	
Male gender, %	75%	62.5%	0.321	100%	83.3%	0.529
GA, wk^a^	34 (33–35)	34 (32–5)	0.785	39 (37.5–39.5)	38.5 (38–40)	0.980
BW, g^b^	1,908 (406.0)	2,064 (470.5)	0.202	3,170 (460.7)	2,860 (455.3)	0.109
LBW, %	95%	79.2%	0.210	11.1%	22.2%	0.636
Cesarean section, %	75%	85.4%	0.498	44.4%	72.2%	0.219
1-min Apgar^a^	9 (6–9)	9 (8–10)	0.437	10 (9–10)	10 (9–10)	0.571
5-min Apgar^a^	9.5 (9–10)	10 (9–10)	0.802	10 (9–10)	10 (8–10)	0.571
Primegravidity, %	40%	27.1%	0.294	44.4%	33.3%	0.683
Primipara, %	65%	35.4%	0.025	44.4%	44.4%	1.000
Feeding before onset, %	55%	66.7%	0.363	88.8%	83.3%	1.000
History of perinatal asphyxia, %	60%	50%	0.452	11.1%	16.7%	1.000
Age of onset, d^a^	3.5 (2–5)	3 (2–4)	0.754	3 (1–5)	2.5 (1–4)	0.792
**Symptom, %**						
Abdominal distension	95%	100%	0.294	100%	88.9%	0.538
Lethargy	80%	83.3%	0.737	88.8%	66.6%	0.363
Shortness of breath	60%	68.8%	0.487	33.3%	55.5%	0.420
Cyanosis	50%	54.2%	0.754	33.3%	55.5%	0.420
Vomiting	35%	20.8%	0.219	66.6%	44.4%	0.420
Feeding intolerance	30%	10.4%	0.102	55.5%	22.2%	0.108
Fever	25%	4.2%	0.033	22.2%	5.6%	0.250
Bloody stool	10%	4.2%	0.714	11.1%	5.6%	1.000
**Signs, %**						
Absent bowel sounds	80%	79.2%	1.000	77.7%	66.6%	0.676
Prominent abdominal veins	50%	31.3%	0.144	33.3%	61.1%	0.236
Abdominal erythema	35%	16.7%	0.180	44.4%	22.2%	0.375

*IQR, interquartile range; GA, Gestational age; BW, Birth weight*.

a *Median and interquartile range*.

b *Mean and standard deviation*.

The laboratory findings and imaging features are shown in Table 7. In pre-term infants, we found abnormal WBC count, thrombocytopenia, and serum lactic acid > 2.5 mmol/L were more frequent among the non-survival group. In term infants, a similar trend was found, but the difference was not statistically significant.

**Table 7:** Laboratory findings and imaging features of pre-term and full-term infants.

	**Pre-term group**			**Term group**		
	**Non-survivors**	**Survivors**	***p-*value**	**Non-survivors**	**Survivors**	***p-*value**
	**(*n* = 20)**	**(*n* = 48)**		**(*n* = 9)**	**(*n* = 18)**	
**Laboratory findings**						
WBC count, 10^9^cells/L^a^	3.33 (2.1–6.2)	8.37 (4.5–12.3)	0.002	9.30 (4.71)	11.57 (6.44)	0.380
<5 or >20, %	85%	45.8%	0.003	33.3%	22.2%	0.653
Hb, g/L^b^	163.51 (35.3)	157.17 (29)	0.442	183.33 (23.2)	160.06 (28.6)	0.055
PLT count, 10^9^cells/L^a^	146 (82–177)	227 (164–281)	<0.0001	197 (146–257)	229 (118–302)	0.918
Thrombocytopenia, %	50%	20.8%	0.016	44.4%	33.3%	0.683
CRP > 8 mg/L, %	45%	41.7%	0.800	77.8%	61.1%	0.667
Higher PCT level, %	83.3%	61.1%	0.097	88.8%	85.7%	1.000
pH^b^	7.16 (0.159)	7.30 (0.129)	<0.0001	7.12 (0.164)	7.32 (0.151)	0.003
pH <7.3, %	75%	52.1%	0.080	88.9%	38.9%	0.019
Lac, mmol/L^a^	4.3 (1.9–7.2)	1.6 (1.1–2.7)	0.001	6.6 (3.3–7.8)	1.8 (1.2–2.9)	0.002
Lac > 2.5 mmol/L, %	70%	31.3%	0.003	88.9%	27.8%	0.004
SC, mEq/L^b^	134.67 (5.7)	136.24 (4.0)	0.272	130.52 (5.8)	137.58 (4.8)	0.002
Hyponatremia, %	25%	8.3%	0.146	44.4%	5.6%	0.030
**Imaging features**						
Pneumoperitoneum, %	100%	97.9%	1.000	100%	83.3%	0.529

*WBC, White blood cell; Hb, Hemoglobin; PLT, platelet; CRP, C-reactive protein; PCT, procalcitonin; Lac, lactic acid; SC, Sodium concentration*.

a *Median and interquartile range*.

b *Mean and standard deviation*.

The most common perforation site was at the curvature major in the pre-term group, followed by the curvature minor. In term group, curvature major and anterior wall were the most common perforation sites. In the two cohorts, the amount of ascites in the non-survival group was higher than in the survival group (Table 8).

**Table 8:** Surgical findings and operative procedures of pre-term and full-term infants.

	**Pre-term group**			**Term group**		
	**Non-survivors**	**Survivors**	***p-*value**	**Non-survivors**	**Survivors**	***p-*value**
	**(*n* = 20)**	**(*n* = 48)**		**(*n* = 9)**	**(*n* = 18)**	
Surgical intervention time, h			0.403			1.000
≤ 24 h, %	75%	64.6%		66.7%	61.1%	
>24 h, %	25%	35.4%		33.3%	38.8%	
Perforation site, %			0.584			0.534
Curvatura major	30%	18.8%		22.2%	38.9%	
Curvatura minor	10%	18.4%		11.1%	0%	
Anterior wall	15%	10.4%		22.2%	22.2%	
Posterior wall	5%	2.1%		0%	0%	
Unspecified	40%	50%		44.4%	38.9%	
Amount of ascites, ml^a^	150 (60–190)	100 (50–150)	0.020	150 (75–200)	100 (50–150)	0.081
<100, %	30%	47.9%	0.173	22.2%	38.9%	0.667
≥100, %	70%	52.1%		77.8%	61.1%	
Multiple perforations, %	35%	18.8%	0.260	11.1%	11.1%	1.000
Surgical procedures, %			0.318			0.250
Gastrorrhaphy+gastrostomy	85%	81.3%		77.8%	94.4%	
Gastrorrhaphy+drainage	10%	18.8%		11.1%	5.6%	
Drainage	5%	0%		11.1%	0%	

a *Median and interquartile range*.

Pneumonia, sepsis, and coagulopathy were the most frequently observed complication in both two cohorts. In pre-term infants, shock and sclerema neonatorum were more common in the non-survival group. In term infants, shock and coagulopathy were more frequent in the non-survival group (Table 9).

**Table 9:** Complications and causes of perforation of pre-term and full-term infants.

	**Pre-term group**			**Term group**		
	**Non-survivors**	**Survivors**	***p-*value**	**Non-survivors**	**Survivors**	***p-*value**
	**(*n* = 20)**	**(*n* = 48)**		**(*n* = 9)**	**(*n* = 18)**	
**Complications, %**						
Sepsis	80%	60.4%	0.120	77.8%	38.9%	0.103
Pneumonia	85%	89.6%	0.685	66.7%	88.9%	0.295
Respiratory failure	45%	50%	0.707	22.2%	22.2%	1.000
Shock	60%	22.9%	0.003	77.8%	33.3%	0.046
Coagulopathy	70%	60.4%	0.455	77.8%	33.3%	0.046
NEC	30%	25%	0.670	33.3%	5.6%	0.093
SN	30%	6.3%	0.016	22.2%	0%	0.103
Cause of the perforation, %			0.346			0.333
Only defects of the gastric muscle wall	20%	31.3%		11.1%	0%	
Other causes	80%	68.8%		88.9%	100%	

*NEC, neonatal necrotizing enterocolitis; SN, sclerema neonatorum*.

In pre-term infants, shock, thrombocytopenia, and serum lactic acid > 2.5 mmol/L remained independent prognostic factors in multivariate logistic regression (Table 10).

**Table 10:** Risk factors associated with mortality in pre-term infants.

	**Univariable OR**	***p-*value**	**Multivariable OR**	***p-*value**
	**(95% CI)**		**(95% CI)**	
Primipara	3.387 (1.135–10.101)	0.029		
Shock	5.045 (1.647–15.456)	0.005	3.522 (1.004–12.351)	0.049
SN	6.429 (1.420–29.104)	0.016		
WBC count <5 or >20 × 10^9^/L	3.889 (1.268–11.928)	0.018		
Thrombocytopenia	3.8 (1.24–11.624)	0.019	4.064 (1.155–14.303)	0.029
Lac > 2.5 mmol/L	5.133 (1.651–15.963)	0.005	3.613 (1.017–12.834)	0.047

*CI, confidence interval; OR, odd ratio; SN, sclerema neonatorum; WBC, White blood cell; Lac, lactic acid*.

In full-term infants, the incidences of shock, coagulopathy, pH < 7.3, hyperlactatemia, and hyponatremia in the non-survival group were higher than in the survival group. Multivariate analysis was not performed due to the small number of events in this cohort (Table 11).

**Table 11:** Risk factors associated with mortality in term infants.

	**Univariable OR**	***p-*value**
	**(95% CI)**	
Shock	7.000 (1.098–44.607)	0.039
Coagulopathy	7.000 (1.098–44.607)	0.039
pH < 7.3	12.571 (1.280–123.48)	0.030
Lac > 2.5 mmol/L	20.800 (2.043–211.792)	0.010
Hyponatremia	13.600 (1.225–151.045)	0.034

*CI, confidence interval; OR, odd ratio; Lac, lactic acid*.

## Discussion

In our study, the total mortality of neonates with gastric perforation was 29.7% (30/101), which was consistent with previous studies (4, 9, 16, 21, 22).

Our study confirmed that the most common presentations at admission were abdominal distension, lethargy, and shortness of breath, which is in line with previous studies (4, 23, 24). Neonatal showed signs of fever, feeding intolerance, or abdominal erythema, often indicating a poor condition, so clinicians should pay more attention to these patients.

Irrespective of etiology, NGP mainly occurs between 2 and 7 days of age (25). The median age of symptom onset was 3 d (range, 1–11 *d*) in our study, which is consistent with other studies (4, 17, 23, 26).

Our study confirmed that serum lactic acid > 2.5 mmol/L was associated with death, which has not been reported in previous studies on NGP. Previous studies (27, 28) have demonstrated that serum lactic acid is a universally accepted, clinically helpful indicator of tissue hypoperfusion. The hypoperfusion pressure prevents nutrient and oxygen supply to peripheral tissue, which may further impede the healing process and damage the integrity of gastrointestinal mucosa. In addition, Rhodes et al. (29) found that patients with persistent elevation of serum lactic acid had a significantly increased risk of refractory shock and multiple organ dysfunction. For these reasons, elevated serum lactic acid indicates a poor prognosis of NGP.

In our study, the shock was a vital variable significantly associated with a poor prognosis of NGP. Shock is often related to circulatory dysfunction, which decreases the supply of oxygen and nutrient to peripheral tissues, thereby hindering wound healing (30). In shock states, blood flow to the brain and heart is maintained due to redistribution of blood away from peripheral organs, which destroys the gut integrity barrier, enhances bacterial translocation, and leads to sepsis and multiple organ dysfunction (31). Therefore, in patients with clinical suspicion of neonatal gastric perforation, signs of hypovolemia or shock must be actively examined because timely treatment of this complication is critical to prognosis.

Our study confirmed that thrombocytopenia was associated with mortality of neonates with gastric perforation, which is consistent with a previous study (17).

Yang et al. (23) reported abnormal white blood cell count was associated with mortality. Iacusso et al. (26) reported that sepsis was the only variable significantly associated with mortality. In our study, abnormal white blood cell count and sepsis were more common in patients with poor prognosis. However, we did not find a statistically significant association between them, which is consistent with a previous investigation (16). This suggested that abnormal white blood cell count and sepsis may not be related to the prognosis of NGP. Therefore, it is necessary to closely integrate clinical symptoms and other laboratory findings to make a correct judgment of patients' condition, even if there is no obvious abnormality of white blood cells or sepsis in patients.

Hyponatremia (serum sodium < 130 mEq/L) and metabolic acidosis (pH < 7.3) have been reported as risk factors for mortality in patients with neonatal gastric perforation (4, 15, 16). However, we found no association between these factors and the prognosis of NGP in our study. The development of critical care has improved our ability to correct metabolic and electrolyte abnormalities before they become irreversible. More than half of patients were diagnosed with hyponatremia or acidosis in our study, and their electrolyte and acid-base disturbances were corrected in time.

Whether pre-term birth and low birth weight are risk factors for mortality of NGP remains controversial (4, 15, 16, 32). In this study, the proportion of pre-mature and low birth weight in the non-survival group was higher than that in the survival group, but no statistical association was found.

Once perforation is confirmed, surgical laparotomy should be performed. Gastrorrhaphy alone or combined with gastrostomy is the most common surgery for NGP. As for the associated gastrointestinal anomalies, concurrent or delayed surgical correction should be decided based on the specific situation of patients. Byun et al. (4) have found that the time between symptoms and surgical intervention was the only prognostic factor for survival, which in the present study we did not. Our results agree with similar results obtained in another study (23).

This study found a marginal statistical association (*p* = 0.068) between sclerema neonatorum (SN) and death. SN is usually caused by cold, pre-term birth, infection, and hypoxia. The development in neonatal intensive care has dramatically reduced the incidence of NS. In this study, only 11 children were diagnosed with SN, and the incidence of SN in the non-survival group and survival group was 26.7 and 4.2%, respectively. We speculated that patients with SN were more likely to have circulatory disturbance and coagulopathy, which increased the possibility of other organ injury and disseminated intravascular coagulation in patients with NGP, leading to a poor prognosis. However, no similar reports have been found in foreign literature, so this conclusion needs to be confirmed further *via* large sample studies.

Due to the differences in the pathogenesis and morbidity of NGP between full-term infants and pre-term infants, it is necessary to explore their prognostic factors separately. In pre-term infants, we found that shock, thrombocytopenia, and serum lactic acid > 2.5 mmol/L were associated with death, which was consistent with those of the overall study population. In term infants, multivariate analysis was not performed due to the small number of events in this cohort. In term infants, the incidence of thrombocytopenia was not significantly different between the non-survival group and the survival group. The main reason for this is probably the sample size, which was not large enough to detect small differences. There have been no similarities in foreign literature, so these findings need to be confirmed in studies with a larger sample size and a multicenter design.

Gastric fundus, curvature major and minor were reported as the most common perforation sites in neonates (33). Our results were consistent with this. In our study, perforations in curvature minor of the stomach seemed to be more likely to occur in pre-term infants. Incoordination of gastroesophageal motility, typical of pre-mature neonates, is considered as a possible mechanism causing increased intragastric pressure, leading to gastric rupture (8). When gastric rupture occurs solely from excessive expansion, the rupture is usually along the curvature minor (34).

The main strength of our study is that it is the largest retrospective cohort study among patients with NGP. And in this study, we examined potential risk factors such as SN, amount of peritoneal fluid, etc.

This study has some limitations. First, this is a retrospective series focused on hospitalized patients. Given that these patients presented with more severe disease, our data may overestimate overall incidences of poor prognosis in the patients with NGP. Second, this is a retrospective study and data might be missing, which may have led to an underestimation of their potential predictive value. Finally, the development in intensive medical care over time undoubtedly affects the overall clinical outcomes and may act as a confounder when we look for prognostic factors of NGP.

## Conclusion

The present study explored the possible prognostic factors of NGP and extended the current understanding with respect to relevant clinical outcomes. In our study, the factors affecting prognosis of NGP were shock, thrombocytopenia, and hyperlactemia (serum lactic acid > 2.5 mmol/L). Although further studies are warranted to investigate whether prevention and control of modifiable risk factors for excess mortality could favorably modify outcomes, our data may help to move to a more comprehensive risk assessment in patients with NGP, and provide the rationale for a strategy of prevention and optimal interventions in the future.

## Data Availability Statement

The raw data supporting the conclusions of this article will be made available by the authors, without undue reservation.

## Ethics Statement

This study was approved by the Ethical Guidance of the Ethical Review Board of Children's Hospital of Chongqing Medical University (2020-289). As private information including patient ID, residence and contact information is crypted and hashed, the review board agreed to waive the statement of consent.

## Author Contributions

YH, YS, QZ, and PY acquired the data. YH, QL, and LW analyzed the data and drafted the manuscript. NP and QL revised the manuscript. All authors contributed to conception and design of the research, interpretation of the results, and edited the manuscript.

## Conflict of Interest

The authors declare that the research was conducted in the absence of any commercial or financial relationships that could be construed as a potential conflict of interest.

## References

[1] Sato M Hamada Y Kohno M Ise K Uchida K Ogata H. Neonatal gastrointestinal perforation in Japan: a nationwide survey. Pediatr Surg Int. (2017) 33:33–41. 10.1007/s00383-016-3985-z 27696212

[2] Pulzer F Bennek J Robel-Tillig E Knüpfer M Vogtmann CJL. Gastric perforation in a newborn. Lancet. (2004) 363:703. 10.1016/S0140-6736(04)15644-4 15001328

[3] Kara CS Ilçe Z Celayir S Sarimurat N Erdogan E Yeker D. Neonatal gastric perforation: review of 23 years' experience. Surg Today. (2004) 34:243. 10.1007/s00595-003-2675-3 14999537

[4] Byun J Kim HK Seung YN Kim SH Jung SE Lee SC. Neonatal gastric perforation: a single center experience. World J Gastrointest Surg. (2014) 6:151–5. 10.4240/wjgs.v6.i8.151 25161763 PMC4143970

[5] Tan CE Kiely EM Agrawal M Brereton RJ Spitz L. Neonatal gastrointestinal perforation. J Pediatr Surg. (2003) 24:888–92. 10.1016/S0022-3468(89)80589-5 2674391

[6] Holgersen LO. The etiology of spontaneous gastric perforation of the newborn: a reevaluation. J Pediatr Surg. (1981) 16:608–13. 10.1016/0022-3468(81)90014-2 7277163

[7] Dupont P Trentesaux AS Guillemin MG Petit T Guillois B. Idiopathic gastric perforation in the newborn. Report of 2 cases. Gastroenterol Clin Biol. (2003) 27:1160–2. 10.1097/00042737-200312000-00020 14770121

[8] Houck WS Griffin JA. Spontaneous linear tears of the stomach in the newborn infant. Ann Surg. (1981) 193:763–8. 10.1097/00000658-198106000-00012 7247521 PMC1345170

[9] Duran R Inan M Vatansever U Aladag N Acunas B. Etiology of neonatal gastric perforations: review of 10 years' experience. Pediatr Int. (2007) 49:626–30. 10.1111/j.1442-200X.2007.02427.x 17875089

[10] Gleason CA Devaskar SU. Avery's Diseases of the Newborn. 9th ed. Philadelphia, PA: Elsevier Saunders (2012).

[11] Miller R. Observations on the gastric acidity during the first month of life. Arch Dis Child. (1941) 16:22. 10.1136/adc.16.85.22 21032189 PMC1987752

[12] Touloukian RJ Posch JN Spencer RJ. The pathogenesis of ischemic gastroenterocolitis of the neonate: selective gut mucosal ischemia in asphyxiated neonatal piglets. J Pediatr Surg. (1972) 7:194–205. 10.1016/0022-3468(72)90496-4 5023196

[13] Shaker IJ Schaefer JA James AE White JJ. Aerophagia, a mechanism for spontaneous rupture of the stomach in the newborn. Am Surg. (1973) 39:619–23. 4746597

[14] Daniel TJS. Spontaneous rupture of the stomach in the newborn: a clinical and experimental study. Surgery. (1965) 58:561. 14338551

[15] Ghung MS Kuo C Wany JW Hsieh WS Huang CB Lin JN. Gastric perforation in the neonate: clinical analysis of 12 cases. Acta Paediatrica Sinica. (1994) 35:460–5. 7942035

[16] Lin CM Lee HC Kao HA Hung HY Hsu CH Yeung CY. Neonatal gastric perforation: report of 15 cases and review of the literature. Pediatr Neonatol. (2008) 49:65–70. 10.1016/S1875-9572(08)60015-7 18947001

[17] Chang-Yo Y Reyin L Ren-Huei F Chu S-M Hsu J-F Lai J-Y. Prognostic factors and concomitant anomalies in neonatal gastric perforation. J Pediatr Surg. (2015) 50:1278–82. 10.1016/j.jpedsurg.2015.04.007 25957026

[18] Goldstein B Giroir B Randolph A. International pediatric sepsis consensus conference: definitions for sepsis and organ dysfunction in pediatrics. Pediatr Crit Care Med. (2005) 6:2–8. 10.1097/01.PCC.0000149131.72248.E6 15636651

[19] Stocker M Fontana M El Helou S Wegscheider K Berger TM. Use of procalcitonin-guided decision-making to shorten antibiotic therapy in suspected neonatal early-onset sepsis: prospective randomized intervention trial. Neonatology. (2010) 97:165. 10.1159/000241296 19776651

[20] Noah Z Budek C De WM Weesemayer DE. Nelson Textbook of Pediatrics. 20th ed. Philadelphia, PA: Elsevier Saunders (2015).

[21] Jr, R. Krasna I. H. Spontaneous' neonatal gastric perforation: is it really spontaneous?. J Pediatr Surg. (2000) 35:1066–9. 10.1053/jpsu.2000.7773 10917298

[22] Ekwunife H Jideofor U Victor M Andrew OJ. Gastrointestinal perforation in neonates: aetiology and risk factors. J Pediatr Surg. (2013) 2:30. 10.47338/jns.v2.42 26023450 PMC4422271

[23] Yang T Huang Y Li J Zhong W Tan T Yu J. Neonatal gastric perforation: case series and literature review. World J Surg. (2018) 42:2668–73. 10.1007/s00268-018-4509-x 29392435

[24] Babayigit A Ozaydin S Cetinkaya M Sander S. Neonatal gastric perforations in very low birth weight infants: a single center experience and review of the literature. Pediatr Surg Int. (2017) 34:79–84. 10.1007/s00383-017-4205-1 29079904

[25] Saracli T Mann M French DM Booker CR Scott RB. Rupture of the stomach in the newborn infant: report of three cases and review of the world literature. Clin Pediatr. (1967) 6:583–8. 10.1177/000992286700601014 6077501

[26] Iacusso C Alessandro B Fabio F Pietro B Francesco M. Pathogenetic and prognostic factors for neonatal gastric perforation: personal experience and systematic review of the literature. Front Pediatr. (2018) 6:61. 10.3389/fped.2018.00061 29670869 PMC5893822

[27] Guyette F Suffoletto B Castillo JL Quintero J Callaway C Puyana J-C. Prehospital serum lactate as a predictor of outcomes in trauma patients: a retrospective observational study. J Trauma. (2011) 70:782–6. 10.1097/TA.0b013e318210f5c9 21610386

[28] Shah A Guyette F Suffoletto B Schultz B Quintero J Predis E. Diagnostic accuracy of a single point-of-care prehospital serum lactate for predicting outcomes in pediatric trauma patients. Pediatr Emerg Care. (2013) 29:715–9. 10.1097/PEC.0b013e318294ddb1 23714761

[29] Rhodes A Evans LE Alhazzani W Levy MM Antonelli M Ferrer R. Surviving sepsis campaign: international guidelines for management of sepsis and septic shock: 2016. Intensive Care Med. (2017) 43:304–77. 10.1007/s00134-017-4683-6 28101605

[30] Andersen LW Mackenhauer J Roberts JC Berg KM Donnino MW. Etiology and therapeutic approach to elevated lactate levels. Mayo Clinic Proc. (2013) 88:1127–40. 10.1016/j.mayocp.2013.06.012 24079682 PMC3975915

[31] Scherer LR. Peptic ulcer and other conditions of the stomach. Pediatric Surgery. 7th ed. Philadelphia, PA: Elsevier Saunder (2012). p. 1029–39.

[32] Chen TY Liu HK Yang MC Yang YN Ko PJ Su YT. Neonatal gastric perforation: a report of two cases and a systematic review. Medicine (Baltimore). (2018) 97:e369. 10.1097/MD.0000000000010369 29702982 PMC5944554

[33] Aydin M Zencirolu A Hakan N Erdoan D Ipek MS. Gastric perforation in an extremely low birth weight infant recovered with percutaneous peritoneal drainage. Turk J Pediatr. (2011) 53:467–70. 10.3109/01942638.2011.619119 21980855

[34] Inouye WY Evans G. Neonatal gastric perforation. a report of six cases and a review of 143 cases. Arch Surg. (1964) 88:471–85. 10.1001/archsurg.1964.01310210145024 14088279

